# Human amnion favours tissue repair by inducing the M1‐to‐M2 switch and enhancing M2 macrophage features

**DOI:** 10.1002/term.2193

**Published:** 2016-07-11

**Authors:** Marta Magatti, Elsa Vertua, Silvia De Munari, Marta Caro, Maddalena Caruso, Antonietta Silini, Mario Delgado, Ornella Parolini

**Affiliations:** ^1^ Centro di Ricerca ‘E. Menni’ Fondazione Poliambulanza–Istituto Ospedaliero Brescia Italy; ^2^ Instituto de Parasitologia y Biomedicina ‘Lopez‐Neyra’ CSIC Granada Spain

**Keywords:** human amnion, conditioned medium, mesenchymal stem/stromal cells, monocyte, M1 and M2 macrophages, immunomodulation, wound healing, regenerative medicine

## Abstract

Human amniotic mesenchymal cells (hAMTCs) possess interesting immunomodulatory properties, making them attractive candidates for regenerative medicine applications. Recent *in vivo* reports argue in favour of an important role for macrophages as targets of hAMTC‐mediated suppression of inflammation and the enhancement of tissue repair. However, a comprehensive study of the effects of hAMTCs and their conditioned medium (CM) on human macrophage differentiation and function is unavailable. In the present study we found that hAMTCs and CM induce the differentiation of myeloid cells (U937 and monocytes) towards macrophages. We then investigated their effects on monocytes differentiated toward pro‐inflammatory M1 and anti‐inflammatory M2 macrophages. Monocytes treated under M1 conditions in the presence of hAMTCs or CMs shifted towards M2‐like macrophages, which expressed CD14, CD209, CD23, CD163 and PM‐2 K, possessed higher phagocytic activity and produced higher IL‐10 and lower pro‐inflammatory cytokines. They were also poor T cell stimulators and Th1 inducers, while they were able to increase activated and naïve suppressive Treg subsets. We show that prostaglandins, and not IL‐6, play a role in determining the M2 activation status. Instead, monocytes treated under M2 conditions in the presence of hAMTCs or CM retained M2‐like features, but with an enhanced anti‐inflammatory profile, having a reduced expression of the co‐stimulatory molecule CD80, reduced phagocytosis activity and decreased the secretion of inflammatory chemokines. Importantly, we provide evidence that macrophages re‐educated by CM improve tissue regeneration/repair in wound‐healing models. In conclusion, we identified new cell targets of hAMTCs and their bioactive factors and here provide insight into the beneficial effects observed when these cells are used in therapeutic approaches *in vivo*. © 2016 The Authors Journal of Tissue Engineering and Regenerative Medicine Published by John Wiley & Sons Ltd.

## 1 Introduction

In recent years, increasing evidence has demonstrated that cells isolated from the amniotic membrane of human term placenta possess interesting immunomodulatory properties that make them an attractive tool for the cellular treatment of immune‐related disorders. Indeed, we and others have demonstrated that human amniotic mesenchymal cells (hAMSCs) and epithelial cells (hAECs), as well as conditioned medium (CM) derived from their culture, possess anti‐inflammatory and anti‐proliferative properties that affect cells of the innate and adaptive immune systems (Parolini *et al.*, 2011). These properties include the *in vitro* ability to suppress T cell proliferation (Li *et al.*, 2005; Wolbank *et al.*, 2007; Magatti *et al.*, 2008; Rossi *et al.*, 2012; Pianta *et al.*, 2015). We found that CM obtained from hAMSC culture affects both central and effector memory T lymphocyte subsets, significantly reduces the expression of markers associated with Th1 and Th17 populations and significantly induces the regulatory T cells compartment (Pianta *et al.*, 2015). Furthermore, not only T lymphocytes but also the *in vitro* proliferation of B cells (Li *et al.*, 2005), the cytotoxicity of natural killer cells (Li *et al.*, 2015) and the migration of neutrophils and macrophages (Li *et al.*, 2005; Tan *et al.*, 2014) have been shown to be affected by amniotic cells and their CM. Moreover, we and others have demonstrated that amniotic cells and their CM impair the development of dendritic cells from primary monocytes (Magatti *et al.*, 2009; Kronsteiner *et al.*, 2011; Banas *et al.*, 2014; Magatti *et al.*, 2015). Interestingly, dendritic cell differentiation was not only blocked at the monocyte level, but skewed toward macrophage‐like cells (Magatti *et al.*, 2015).

Macrophages are key players in orchestrating the host innate and adaptive immune responses. They are a heterogeneous population of cells that are strategically distributed throughout the body to patrol the organism, looking for signs of tissue damage or foreign invaders, and participate in each phase of wound repair (Italiani and Boraschi, 2014). Due to their ability to respond to a milieu of different micro‐environmental cues, several macrophage subsets with different phenotypes and functions have been described (Mosser and Edwards, 2008). Although an oversimplification, polarized macrophages are often referred to as M1 for pro‐inflammatory macrophages and as M2 for macrophage activation other than M1 (Mantovani *et al.*, 2004). M1 macrophages develop in response to inflammatory factors such as interferon‐*γ* (IFN*γ*), lipopolysaccharide (LPS) and tumour necrosis factor‐*α* (TNF*α*) and are characterized *in vitro* by the expression of the chemokine receptor CCR7 (CD197) and high levels of the co‐stimulatory molecules CD80 and CD86, resulting in efficient antigen presentation capacity. Moreover, M1 cells possess a interleukin‐(IL)‐12^hi^IL–23^hi^IL–10^lo^ phenotype and produce large amounts of pro‐inflammatory cytokines and chemokines, including TNF, chemokine (C–X–C motif) ligand 9 (CXCL9), CXCL10 and CXCL11 (Mantovani *et al.*, 2004). Instead, M2 macrophages, produce fewer pro‐inflammatory cytokines, have high phagocytic activity and play an important role in the resolution of inflammation and tissue remodelling and repair. Different subtypes of M2 macrophages are generated in response to Th2‐related cytokines (IL‐4, IL‐13 or IL‐4 + LPS; termed M2a, alternative activated or wound‐healing macrophages), Fc*γ* receptors and Toll‐like receptor (TLR) agonists, immune complexes (M2b, or type II macrophages), IL‐10, TGF*β* and glucocorticoids (M2c, or de‐activated macrophages) and TLR and adenosine A2 A receptor (A2AR) agonists (M2d, or angiogenic macrophages) (Ferrante and Leibovich, 2012). *In vitro*, M2 express CD14, CD23 (Fc*γ*–RII), the scavenger receptors CD163 and CD204, the mannose receptor CD206, and CD209 (DC‐SIGN). Furthermore, M2 macrophages are characterized by a IL‐12^lo^IL‐23^lo^IL‐10^hi^ phenotype and possess a variable capacity to produce inflammatory cytokines, depending on the signal utilized for activation (Mosser and Edwards, 2008; Italiani and Boraschi, 2014).

Interestingly, recent reports argue in favour of an important role of macrophages as targets of the amniotic membrane‐derived cell suppression and mediators of resolution of inflammation and enhancement of tissue repair *in vivo*, such as in models of liver fibrosis (Manuelpillai *et al.*, 2012), multiple sclerosis (Liu *et al.*, 2012) and lung fibrosis (Murphy *et al.*, 2012; Cargnoni *et al.*, 2014; Tan *et al.*, 2014). Moreover, while the infiltrating macrophages in control animals were predominantly M1, the predominant macrophage phenotype in amniotic (hAEC)‐treated animals was M2 (Tan *et al.*, 2015, 2014). Interestingly, hAECs required normal host macrophage function to exert their reparative effects (Murphy *et al.*, 2012) and polarization of macrophages from M1 to M2 required hAECs and Tregs (Tan *et al.*, 2015), suggesting that interactions between hAECs, macrophages and T cell subsets are central to lung repair (Tan *et al.*, 2015).

While these *in vivo* studies clearly support the immunomodulatory actions of amniotic membrane‐derived cells on macrophages, an extensive characterization of the effects of the amniotic membrane‐derived cells and their CMs on human macrophage differentiation, polarization and function *in vitro* has yet to be described, and was the aim of the present study. Importantly, the subsequent biological functions of differentiated cells and, specifically, the interaction between macrophages and T cells, were also investigated.

Amniotic cells used in this study were obtained from the mesenchymal region of the amniotic membrane after cell isolation, without any culture passages. To distinguish these cells from hAMSCs obtained after cell passages, herein we refer to them as human amniotic mesenchymal tissue cells (hAMTCs), as previously described (Magatti *et al.*, 2008). We focused the investigation on the role of hAMTCs‐secreted factors and cultured hAMTCs in a transwell system, thus circumventing cell–cell contact. In parallel, we used conditioned medium (CM) from the culture of unstimulated hAMTCs to study whether activating stimuli or crosstalk with immune cells are required for hAMTCs to exert their effect. We have previously shown that IL‐6 and prostanoids (mainly PGE2) are partially responsible for the inhibition of T cell proliferation induced by CM (Rossi *et al.*, 2012). Here, we investigated whether prostanoids and IL‐6 are also involved in mediating the effect of CM on macrophage differentiation.

We mimicked monocyte‐to‐macrophage differentiation *in vitro* using both the U937 myeloid cell line and primary blood monocytes. In addition, primary monocytes were cultured under polarization conditions towards both pro‐inflammatory (herein termed M1 macrophage‐like; obtained with GM‐CSF, IFN*γ*, LPS) and anti‐inflammatory (herein termed M2 macrophage‐like; obtained with M‐CSF, IL‐4, LPS) cells. Macrophage‐like cells obtained in the presence or absence of hAMTCs and CM were analysed for the expression of a broad panel of surface markers, phagocytic ability, production of cytokines/chemokines and ability to stimulate T cell proliferation and polarization. Finally, we also studied the functional properties of macrophages re‐educated by CM and demonstrated their capacity to improve tissue regeneration/repair in both *in vitro* and *in vivo* wound‐healing models.

## 2 Materials and methods

### 2.1 Ethics statements

Human term placentae (*n* = 30) were collected from healthy women after vaginal delivery or caesarean section at term. Peripheral blood was collected from healthy adult donors (*n* = 21). Samples were collected after obtaining informed written consent, according to the guidelines set by the Ethical Committee of the Catholic Hospital (CEIOC) and after their authorization to use placentae for experimental research (Document *Parere 16*/*2012*).

C57Bl/6 mice were purchased from Charles River. All experiments were performed according to the institutional guidelines for the care and use of laboratory animals in research and with the approval of the local committee in the Consejo Superior de Investigaciones Cientificas.

### 2.2 Isolation of human amniotic mesenchymal tissue cells

Placentae were processed immediately after collection. Human amniotic mesenchymal tissue cells (hAMTCs) were isolated from the mesenchymal region of the amniotic membrane as previously described (Soncini *et al.*, 2007; Rossi *et al.*, 2012). hAMTCs were used after isolation. The immunophenotype of freshly isolated hAMTCs, as obtained from FACS analysis, was CD90 (86 ± 12%), CD13 (91 ± 2%), CD73 (52 ± 7%), CD44 (57 ± 12%), CD105 (8 ± 7%), CD14 (10 ± 2%), HLA‐DR (8 ± 2%), as previously reported (Magatti *et al.*, 2015).

### 2.3 Preparation of conditioned medium from hAMTCs

To obtain conditioned medium (CM) generated from hAMTCs, freshly isolated hAMTCs were cultured for 3 days in 24‐well plates (Corning, NY, USA) at a density of 3 × 10^5^ cells/well in 0.5 ml Roswell Park Memorial Institute (RPMI) complete medium, composed of RPMI 1640 medium (Sigma‐Aldrich, St Louis, MO, USA) supplemented with 10% heat‐inactivated fetal bovine serum (FBS; Sigma‐Aldrich), 2 mm l‐glutamine (Sigma‐Aldrich) and 100 U/ml penicillin and 100 mg/ml streptomycin (pen–strep, herein referred to as P/S; both from Sigma‐Aldrich).

To obtain CM without prostaglandins (CM – PG), hAMTCs were cultured as described above in the presence of 10 μm indomethacin (Sigma‐Aldrich), a cyclooxygenase inhibitor. PGE2 quantification in CM and CM – PG was obtained using a Prostaglandin E2 EIA Kit (Cayman Chemical Co., Ann Arbor, MI, USA), according to the manufacturer's instructions. Absorbance was measured at 405 nm using a microplate reader.

At the end of the culture period, CM and CM – PG were collected, centrifuged at 300 × *g*, filtered through a 0.8 μm sterile filter (Sartorius Stedim, Florence, Italy) and kept frozen at −80 °C until use.

To obtain results that were least influenced by single donor variability and more representative of soluble factors released by hAMTCs, 10 pools, each containing CM from three to six different cell preparations, were used.

### 2.4 Generation of U937‐derived macrophage‐like cells

The human promonocytic cell line U937 (originally isolated from a patient with histiocytic lymphoma) was obtained from the Centro Substrati Cellulari, Istituto Zooprofilattico (Brescia, Italy) and maintained in RPMI complete medium at 37 °C and 5% CO_2_.

To induce macrophage differentiation, 0.5 × 10^6^ U937 cells were cultured in 24‐well plates (Corning) at 37 °C for 3 days in 1 ml RPMI complete medium containing 130 nm phorbol 12‐myristate 13‐acetate (PMA; Sigma‐Aldrich).

### 2.5 Generation of monocyte‐derived M1 and M2 macrophage‐like cells

To obtain monocytes, human peripheral blood mononuclear cells (PBMCs) were separated through density gradient centrifugation (Histopaque, Sigma‐Aldrich) of heparinized buffy coats. Monocytes were isolated from PBMC (*n* = 11) by positive selection, using anti‐CD14‐coated microbeads and MACS® separation columns (Miltenyi Biotec, Bergisch Gladbach, Germany), according to the manufacturer's instructions.

Monocyte‐derived macrophage‐like cells were generated following protocols based on the use of granulocyte‐macrophage colony‐stimulating factor (GM‐CSF) for M1 differentiation and macrophage colony‐stimulating factor (M‐CSF) for M2 differentiation (Verreck *et al.*, 2004; Durafourt *et al.*, 2012). In detail, to obtain fully polarized (herein termed M1) macrophage‐like cells, 0.5 × 10^6^ monocytes were cultured in 24‐well plates at 37 °C in 1 ml RPMI complete medium containing 5 ng/ml human recombinant GM‐CSF (Miltenyi Biotec) for 4 or 5 days, and subsequently supplemented with 20 ng/ml interferon‐*γ* (IFN*γ*; BD Biosciences) for 1 h and then with 100 ng/ml lipopolysaccharide (LPS; Sigma Aldrich) for 48 h. Meanwhile, to obtain herein termed M2 macrophage‐like cells, 0.5 × 10^6^ monocytes were cultured in 24‐well plates at 37 °C in 1 ml RPMI complete medium containing 20 ng/ml M‐CSF (Miltenyi Biotec) for 4 or 5 days and subsequently supplemented with 20 ng/ml interleukin‐4 (IL‐4; kindly provided by Dr Schweighoffer, Novartis, Vienna, Austria) for 1 h and then with 100 ng/ml LPS (Sigma‐Aldrich) for 48 h.

### 2.6 Exposure of U937 cells or monocytes to hAMTCs or CM‐hAMTCs

Co‐cultures of U937 cells or CD14^+^ monocytes with hAMTCs were established with physical separation using transwell chambers in either the absence or presence of macrophage differentiation conditions. Specifically, 0.25 × 10^6^ hAMTCs were plated in the upper compartment of a transwell (0.4 μm pore, polycarbonate membrane, Corning) of 24‐well plates. The following day, U937 cells or monocytes were plated in the lower compartment in a final volume of 1 ml RPMI complete medium. In the case of U937 cells, 0.25 × 10^6^ U937 cells were plated (U937:hAMTCs ratio of 1:1) in the absence of macrophage differentiation conditions, and 0.5 × 10^6^ U937 cells (ratio of 2:1) under macrophage differentiation conditions. In the case of CD14^+^ monocytes, 0.5 × 10^6^ monocytes (monocyte:hAMTCs ratio of 2:1) were plated in either the absence or presence of macrophage differentiation conditions. Macrophage differentiation was performed as described in Section 2.5.

For experiments with CM, U937 cells or monocytes were cultured in 24‐well plates together with 0.5 ml CM (in a final volume of 1 ml) in the absence or presence of a differentiation stimulus, as described above. In addition, M1 differentiation was performed in the presence of 0.5 ml CM – PG or, in order to block the activity of IL‐6 present in the CM, in the presence of 0.5 ml CM with neutralizing antibodies against IL‐6 (1 μg/ml; BD Biosciences, San Jose, CA, USA), IL‐6 receptor‐*α* (1 μg/ml; R&D Systems, Minneapolis, MN, USA) and gp130 (1 μg/ml; R&D Systems) (herein defined as CM–IL‐6).

### 2.7 U937 cells or monocytes phenotype analysis

U937 cells or monocytes were collected after culture (as described in Section 2.6), by gentle scraping. Cell phenotype was investigated by flow‐cytometry analysis. For surface marker analysis, cells were washed with FACS buffer [0.02% sodium azide (Sigma‐Aldrich) and 0.1% bovine serum albumin (BSA; Sigma‐Aldrich) in PBS], and then incubated for 20 min at 4 °C with anti‐human conjugated monoclonal antibodies or isotype‐matched controls, together with 20 mg/ml polyglobin (Gammagard®, Baxter, IL, USA) prepared in PBS with 1% BSA to block non‐specific binding. After washing in FACS buffer, cells were acquired and analysed using a FACSCalibur and CellQuest Software (BD Biosciences). Dead cells were gated out by iodide propidium (IP, 0.1 μg/ml; Sigma Aldrich) staining.

For the intracellular staining of macrophage markers, cells were fixed and permeabilized with Cytofix/cytoperm solution (BD Biosciences) for 20 min at 4 °C. After washing with 1× BD perm/wash buffer solution (BD Biosciences), cells were stained for 20 min at 4 °C with specific antibody, conjugated using a Zenon® AlexaFluor® 647 Mouse IgG1 Labeling Kit (Invitrogen, Carlsbad, CA, USA), according to the manufacturer's instructions. The cells were then washed with 1× BD perm/wash buffer solution and acquired and analysed using a FACSCalibur and CellQuest Software (BD Biosciences). Dead cells were gated out by side scatter (SSC) and forward scatter (FSC) gating.

The dilutions, clones and suppliers of the antibodies used in this study were as follows: CD1a FITC (dilution 1:150, clone HI149); CD11b PE (1:100, ICRF44); CD11c FITC (1:100, KB90); CD14 PE (1:250, M*φ*P9); CD23 PE (1:50, M‐L233); CD80 FITC (1:100, L307.4); CD86 PE (1:200, 2331, FUN‐1); CD163 PE (1:50, GHI/61); CD197 AlexaFluor® 647 (1:100, 3D12); CD206 FITC (1:50, 19.2); CD209 APC (1:250, DCN46) and macrophage marker (1:1000, PM‐2 K). All the antibodies were purchased from BD Biosciences except for CD11c (Merck Millipore, Billerica, MA, USA), and macrophage marker PM‐2 K (Acris Antibodies, Herford, Germany). A mix of FITC‐ and PE‐conjugated mouse IgG1 (from BD Biosciences), FITC‐PE‐ and APC‐conjugated mouse IgG2b (Biolegend, San Diego, CA, USA) and AlexaFluor‐647‐conjugated rat IgG2a (Serotec, Oxford, UK) served as isotype‐matched negative controls.

### 2.8 Phagocytosis assay

Phagocytosis was measured by cellular uptake of fluorescent yellow‐green latex beads, and was quantified by flow cytometry. In detail, U937 cells obtained after culture with or without differentiation stimuli, or monocytes with differentiation stimuli, in the absence or presence of hAMTCs, CM, CM – PG or CM–IL‐6 (as described in Section 2.6), were incubated in RPMI complete medium containing carboxylate‐modified polystyrene and fluorescent yellow‐green latex beads (1 μm mean particle size; 0.5 μl 2.5% solids/1 ml; Sigma‐Aldrich) for 6 or 24 h at 37 °C. In parallel, to establish a baseline measurement of particle adherence to macrophage‐like cells, we tested the same conditions for the same incubation period at 4 °C. The cells were then washed twice with cold PBS and bead uptake was analysed by flow cytometry, using a FACS Calibur and CellQuest Software (BD Biosciences). Dead cells were gated out by IP (0.1 μg/ml; Sigma‐Aldrich) staining.

### 2.9 Macrophage cytokine/chemokine analysis

Cytokine/chemokine levels were measured in supernatants collected from differentiated monocytes, in the absence or presence of hAMTCs, CM, CM – PG or CM–IL‐6 (as described in Section 2.6). Supernatants from hAMTCs cultured alone in the transwell, or in the presence of the factors used to induce macrophage differentiation, along with the CM, CM – PG and CM–IL‐6 used to treat monocytes, were all included as additional controls (see supporting information, Figure S1). Each supernatant was collected, stored at −80 °C and thawed just before use in cytokine/chemokine assays.

A multiplex bead‐based immunoassay (BD CBA Flex Set system from BD Biosciences) was used to determine the levels of human IL‐1*α*, IL‐1*β*, IL‐8, IL‐10, IL‐12p70, interferon‐inducible protein 10 (IP‐10/CXCL10), monocyte chemoattractant protein 1 (MCP‐1/CCL2), monokine induced by IFN*γ* (MIG/CXCL9), macrophage inflammatory protein (MIP)‐1*α*, MIP‐1*β*, the CC chemokine regulated upon activation, normal T cell expressed and secreted (RANTES/CCL5) and TNF*α*. Samples were processed according to the manufacturer's instructions, acquired using a FACSAria (BD Biosciences) and analysed using FCAP Array software (BD Biosciences).

### 2.10 T cells and macrophages co‐culture

T lymphocytes were purified from PBMC (*n* = 10) through an indirect magnetic labelling system, using the Pan T cell Isolation Kit (Miltenyi Biotec) according to the manufacturer's instructions.

Monocytes differentiated towards M1 or M2 macrophage‐like cells, in the absence or presence of hAMTCs, CM, CM – PG or CM–IL‐6, were harvested by gentle scraping, washed in RPMI complete medium and used as stimulators of alloreactive T cells. Specifically, 5 × 10^3^ irradiated (30 Gy) stimulators were seeded with 1.5 × 10^5^ responder T cells in round‐bottomed 96‐well tissue culture plates (Corning), in a volume of 150 μl RPMI complete medium. After 5 days of co‐culture, T cell proliferation, T cell cytokines and T cell phenotype analysis were assessed as described below.

### 2.11 T cell proliferation assays

Responder T cells were cultured with stimulator macrophages, as described above. After 5 days of co‐culture, T cell proliferation was assessed by adding [^3^H]‐thymidine (0.67 μCi/well; Perkin Elmer, Life Sciences, Zaventem, Belgium) for 16–18 h. The cells were then harvested using a Filtermate Harvester (Perkin‐Elmer) and thymidine incorporation was measured using a microplate scintillation and luminescence counter (Top Count NXT, Perkin Elmer).

### 2.12 T cell cytokine analysis

T cells were cultured with macrophages, as described in Section 2.10. After 5 days of co‐culture, the T cells were stimulated with 25 ng/ml PMA (Sigma‐Aldrich) plus 1 ***μ***g/ml Ionomycin (Sigma‐Aldrich) for 6 h. After 1 h of incubation, 3 ***μ***g/ml Brefeldin A (Sigma‐Aldrich) was added to block cytokine secretion. At the end of the stimulation period, T cells were incubated with 0.5 mm EDTA (Sigma‐Aldrich) for 5 min at room temperature. To improve the efficiency of gating live cells and to decrease non‐specific staining of dead cells, the samples were stained with Zombie NIR Live/Dead Cell Kit (eBiosciences, San Diego, CA, USA) before fixation. Cell fixation was performed with 1× FACS Lysing Solution (BD Biosciences) for 10 min. Subsequently the cells were treated with Cytofix/cytoperm solution (BD Biosciences) for 20 min at 4 °C. After washing with 1× BD perm/wash buffer solution (BD Biosciences), cells were stained with anti‐human CD3 FITC (1:50, UCHT1), CD4 BV421 (1:50, RPA‐T4), CD4 PerCP‐Cy5.5 (1:80, RPA‐T4), CD8 BV510 (1:100, SK1), IL‐4 PE (1:500, MP4‐25D2), IL‐4 BV421 (1:300, 8D4–8), IL‐13 PE (1:50, JES10‐5 A2), INFγAPC (1:3000, B27) and Granzyme‐B PE‐CF594 (1:150, GB11) monoclonal antibodies (all from BD Biosciences except for anti‐IL‐13, which was from Miltenyi Biotec) for 30 min at room temperature. After further washing in 1× BD perm/wash buffer solution, cells were acquired with a FACSAria (BD Biosciences) and analysed with FCS express v. 4.07 (DeNovo Software, Los Angeles, CA, USA). The analysis was restricted to gated CD4^+^ and CD8^+^ T cells. The gating strategy is shown in Figure S2A (see supporting information).

### 2.13 T cell phenotype analysis

T cells were cultured with macrophages as described above. After 6 days of co‐culture, T cell phenotype was investigated by flow cytometry. Samples were stained with Zombie NIR Live/Dead Cell Kit (eBiosciences, San Diego, CA, USA) and then fixed with 0.5% methanol‐free formaldehyde (ThermoFisher, Waltham, MA, USA) for 15 min at room temperature (RT). The surface staining was carried out for 30 min at RT, using the following anti‐human antibodies: CD4 BV421 (1:50, RPA‐T4), CD45RA FITC (1:80, HI100) or CD45RA PerCP‐Cy5.5 (1:80, HI100), CD25 PerCP‐Cy 5.5 (1:40, M‐A251) or CD25 BV421 (1:100, M‐A251), CD119 PE (1:80, GIR‐208), CD183 PE‐Cy7 (1:40, 1C6/CXCR3). Cells were then permeabilized with 0.05% Saponin/100 mm Tris–HCl, pH 7.4, for 15 min. The staining of intracellular antigens was performed by incubating cells for 30 min with anti‐human antibodies FoxP3 PE‐CF594 (1:30, 259D/C7) or GATA3 AlexaFluor 647 (1:20, L50–823; both from BD Biosciences). Cells were acquired with a FACSAria (BD Biosciences) and analysed using FCS express v. 4.07 (DeNovo Software). Gating strategies are shown in Figures S2B (Th1 and Th2) and S2C (Treg) (see supporting information).

### 2.14 *In vitro* scratch wound assay

Fibroblasts were isolated and cultured as previously described (Magatti *et al.*, 2012). For *in vitro* scratch wound assay, fibroblasts were plated in 24‐well plates (40 000 cells/cm^2^ = 80 000 cells/well), and scratched with a 10 μl micropipette tip the day after. After scratching, the area of each wound was measured using ImageJ software (time 0). Then, M1, M1_CM, M2 (10 000 cells/well) or CM (100 μl/well) were plated in co‐culture with fibroblasts in a final volume of 1 ml/well RPMI complete medium; 22 h after scratching (T22), the amount of wound remaining was measured and the remaining unmigrated area of each well was calculated, considering the largest scratch area measured at T0 as 100%, as follows:

$$100–\left[\left(\text{wound}\;{\text{area}}_{T0}–{\text{wound area}}_{T22}\right)\times 100/\left(\text{largest}\;{\text{scratch area}}_{T0}\right)\right].$$

Cell morphology allowed us to clearly distinguish fibroblasts (elongated cells) from macrophages (smaller, rounded cells). In order to ensure that the measurement of the unmigrated area was not affected by the presence of macrophages, 22 h after scratch, the cell cultures were washed in PBS (Sigma‐Aldrich), fixed in 2% methanol‐free formaldehyde solution (ThermoFisher) for 20 min and stained immunocytochemically with anti‐human CD45 antibody (dilution 1:400, clone 2D1). Endogenous peroxidase activity was blocked using Dako Real solution (Dako‐Cytomation, Glostrup, Denmark) for 10 min, while unspecific binding was blocked with 2.5% normal horse serum (Vector Laboratories, Burlingame, CA, USA) for 30 min. The primary antibody was then applied for 1.5 h at room temperature, followed by incubation in ImmPRESS™ (Peroxidase) Polymer Anti‐Mouse IgG (rat adsorbed) reagent (Vector Laboratories) for 30 min. 3,3‐Diaminobenzidine (Vector Laboratories) was used as peroxidase substrate solution, and haematoxylin and eosin (H&E; Bio Optica Milano S.p.A, Milan, Italy) was used for counterstaining.

### 2.15 Mouse macrophages isolation and culture

Murine macrophages were generated from C57Bl/6 bone marrow. Briefly, bone marrow cells (4 × 10^5^/ml) were cultured in complete Dulbecco's modified Eagle's medium (DMEM; 2 mm l‐glutamine, 100 U/ml P/S and 20% heat‐inactivated FCS, all from Gibco/Invitrogen) containing 20 ng/ml M‐CSF (Peprotech) for 7–8 days. Differentiated macrophages were detached by incubating the plates with 2 mm EDTA/PBS at 37 °C for 10 min. Cell preparations generally consisted of >95% CD11b^+^ CD11c^−^ macrophages. Bone marrow‐derived macrophages were plated at 8 × 10^5^ cells/well in six‐well plates. After 4 h of adherence, the macrophages were washed with PBS and stimulated with IFN*γ* (BD Bioscience; 10 ng/ml) and LPS (Sigma; isotype B4:11, 1 μg/ml) in 5 ml complete DMEM (control) or in 2.5 ml complete DMEM mixed with 2.5 ml CM for 48 h. Culture supernatants were collected and assayed for cytokine contents by specific sandwich ELISAs, using capture/biotinylated detection antibodies from BD Pharmingen, according to the manufacturer's recommendations, and for nitrite contents by using the Griess assay, as previously described (Anderson *et al.*, 2013). Macrophages were detached as described above and used for treating skin wounds in diabetic mice.

### 2.16 Model of skin wound in diabetic mice

Diabetes was induced in 8 week‐old mice by a single intraperitoneal injection of 100 mg/kg Streptozotocin (STZ; Sigma) in citrate buffer, pH 4.5. Blood glucose levels were measured using a glucometer (Accutrend Plus, Roche) 4 weeks after treatment with STZ to confirm successful induction of diabetes. Animals with blood glucose levels >400 mg/dl were considered diabetic and were used to perform skin wounds. The mice were anaesthetized, the dorsal hair was shaved and full‐thickness surgical skin wounds 6 mm in diameter were made aseptically on either side of the dorsal midline. The mice were intraperitoneally injected with untreated or CM‐treated macrophages (10^6^ cells/mouse, once, 6 h after injury) or subcutaneously injected on the back with CM (200 μl/injection) immediately, 2 days and 4 days after surgery. The wounds were digitally photographed every day for 2 weeks and the wound areas were calculated from the photographs using ImageJ image analysis software.

### 2.17 Determination of gene expression in skin wounds

Total RNA was isolated using Tripure (Roche) from skin samples collected 6 days after surgery. After DNase I treatment, RNA (1 μg/sample) was reverse‐transcribed using a Revert Aid First Strand cDNA Synthesis kit (Fermentas) and random hexamer primers. Real‐time quantitative PCR (60 °C as annealing temperature) was performed, using iQ SYBR Green Supermix (Bio‐Rad), according to the manufacturer's instructions, using *β*‐actin for normalization and estimating fold change expression using the ∆∆*C* t method. The primer pairs were: Fizz1, forward 5′‐ACTTGTTCCCTTCTCATCTG‐3′, reverse 5′‐TCCACCTCTTCATTCTTAGG‐3′; TNF*α*, forward 5′‐CCCTCACACTCAGATCATCTTCT‐3′, reverse 5′‐GCTACGACGTGGGCTACAG‐3′; iNOS, forward 5′‐GTTCTCAGCCCAACAATACAAGA‐3′, reverse 5′‐GTGGACGGGTCGATGTCAC‐3′; TGF*β*1, forward 5′‐TGCGCTTGCAGAGATTAAAA‐3′, reverse 5′‐AGCCCTGTATTCCGTCTCCT‐3′; SMA, forward 5′‐AGGGAGTAATGGTTGGAATGG‐3′, reverse 5′‐TGATGATGCCGTGTTCTATCG‐3′; CD31, forward 5′‐ACTTCTGAACTCCAACAGCGA‐3′, reverse 5′‐CCATGTTCTGGGGGTCTTTAT‐3′; VEGF, forward 5′‐GAGAGAGGCCGAAGTCCTTT‐3, reverse 5′‐TTGGAACCGGCATCTT TATC‐3′; *β*‐actin, forward 5′‐AATCGTGCGTGACATCAAAG‐3′, reverse 5′‐ATGCCACAGGATTCCATACC‐3′.

### 2.18 Statistical analysis

For each parameter investigated, two‐way analysis of variance (ANOVA) was used to evaluate the effect of different treatments (hAMTCs, CM) and differentiation conditions applied (monocytes, M1, M2). Multiple comparison were adjusted by the appropriate Tukey's method. Moreover, to test our hypothesis that hAMTCs or CM drives the differentiation of U937 and M1 cells towards macrophages (U937 + PMA‐like cells) and M2‐like cells, respectively, we implemented the analysis with multiple comparisons (using the Holm–Sidak method) to compare hAMTC‐ or CM‐treated U937/M1 cells with U937 + PMA and M2‐macrophage‐like cells; *p* < 0.05 was considered statistically significant. Statistical analysis was performed using Prism 6 (GraphPad Software, La Jolla, CA, USA).

## 3 Results

### 3.1 Exposure to hAMTCs or CM induces U937 cells to differentiate towards macrophage‐like cells

U937 is a myeloid cell line that can be induced with PMA to differentiate to macrophages (Minta and Pambrun, 1985), expressing surface markers including CD11b and CD11c subunits of the *β*2‐integrin family, the antibody receptor CD23 (Fc*γ*‐RII) and the co‐stimulatory molecule CD86 (B7.2) (see supporting information, Figure S3A). It is noteworthy that U937 cells co‐cultured in transwell with hAMTCs or exposed to CM (in the absence of PMA) had increased expression of the macrophage‐associated markers CD11b, CD11c, CD23, CD86 and CD14 (see supporting information, Figure S3A), indicating that hAMTCs and CM induced macrophage‐like differentiation of U937 cells. In fact, multiple comparison analysis did not reveal significant differences between markers expressed by hAMTCs‐ or CM‐treated U937 vs PMA‐treated U937 cells. Similar to PMA treatment, hAMTCs and CM did not affect the expression of other markers, such as CD1a, CD197, CD80, CD209 or CD163 (see supporting information, Figure S3A). Moreover, hAMTCs and CM did not change the expression of these factors in PMA‐activated U937 cells, with the exception of CD23, CD86 and CD11b, which were further increased (data not shown).

Because phagocytosis is a characteristic of differentiated macrophages, we assayed the capacity of these cells to engulf fluorescent latex beads. As expected, treatment with PMA, hAMTCs or CM increased the percentage of U937 cells able to phagocytose and their respective phagocytic activity (measured as the mean of fluorescence intensity in each cell) (see supporting information, Figure S3B). Taken together, these results suggest that hAMTCs induce the differentiation of U937 monocytes into cells that are macrophage‐like in both phenotype and function.

### 3.2 Exposure to hAMTCs or CM induces human monocytes to differentiate towards M2 macrophage‐like cells

**Figure 1:**
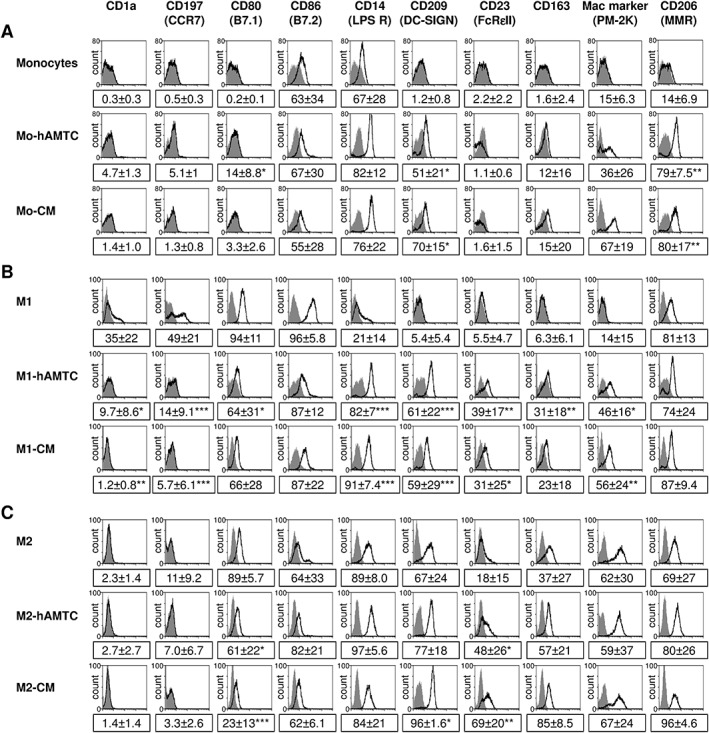
Effect of hAMTCs and CM on phenotype and differentiation of monocytes towards M1 and M2 macrophage‐like cells. Phenotype of CD14^+^ monocytes cultured in (A) naïve, (B) M1 or (C) M2 (C) differentiation conditions in the absence (control monocytes, M1 and M2) or presence of hAMTCs (Mo‐hAMTCs, M1‐hAMTCs and M2‐hAMTCs) or CM (Mo‐CM, M1‐CM and M2‐CM). Flow cytometry was performed by incubating cells with specific anti‐human monoclonal antibodies (white histograms) or isotype‐matched IgGs (control, grey histograms). The histograms shown are representative of at least three (A) or four (B, C) individual experiments; the numbers represent mean ± SD of the percentage of positive cells for each marker (*p < 0.05, **p < 0.01, ***p < 0.001 vs control monocytes, M1 or M2)

Based on the observation that hAMTCs and CM induce macrophage‐like differentiation of U937 cancer cells, we then investigated the effect of hAMTCs and CM on human peripheral blood monocytes. The presence of hAMTCs or CM in human monocyte cultures increased the surface expression of the macrophage mannose receptor (MMR, CD206) and the macrophage‐specific antibody (clone PM‐2K) (Figure 1A), indicative of differentiation towards macrophages (Takeya *et al.*, 1991, Martinez‐Pomares, 2012). Interestingly, monocytes exposed to hAMTCs or CM expressed CD209 (DC‐SIGN) and CD163 (Figure 1A), markers generally attributed to M2 macrophage‐like cells (Mantovani *et al.*, 2004).

Therefore, we next investigated the effects of hAMTCs and CM on macrophage polarization. In line with other reports (Verreck *et al.*, 2006), primary monocytes differentiated towards M1 macrophage‐like cells showed an adherent rounded shape, increased expression of CD206, co‐stimulatory molecules CD80 and CD86, chemokine receptor CCR7 (CD197) and decreased/absent expression of CD1a, CD14, CD209, CD23 and CD163 (Figure 1B). Meanwhile, monocytes differentiated toward M2 macrophage‐like cells had an adherent stretched/elongated, spindle‐like morphology and expressed high levels of CD14, CD209, CD23, CD163 and clone PM‐2K (a distinctive marker of M2 macrophages under our culture conditions) and low levels of CD80 and CD86, and were negative for CD1a and CD197 (Figure 1C). Interestingly, polarization of monocytes under M1 conditions in the presence of hAMTCs (M1‐hAMTCs) or their CM (M1‐CM) resulted in M2 macrophage‐like cells, as shown by their expression of CD14, CD209, CD23, CD163 and PM‐K2 and reduced expression of CD80, CD86, CD197 and CD1a (Figure 1B). Multiple comparison analysis did not reveal significant differences between markers expressed by hAMTCs or CM‐treated M1 vs control M2 macrophage‐like cells. These findings suggest that hAMTCs and CM were able to switch monocyte differentiation towards M2 macrophage‐like cells, even in the presence of M1 polarizing conditions. In contrast, the addition of hAMTCs (M2‐hAMTCs) or CM (M2‐CM) to monocyte cultures under M2‐differentiation conditions did not significantly alter M2 macrophage‐like markers, with the exception of increased CD209 and CD23 and decreased CD80 (Figure 1C).

**Figure 2:**
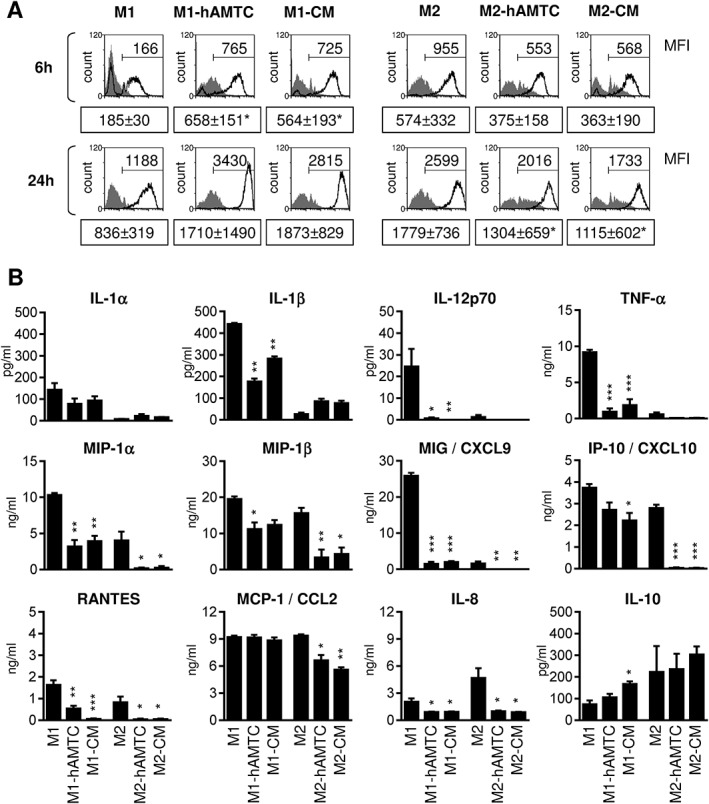
Effect of hAMTCs and CM on phagocytosis and cytokine/chemokine production of M1 and M2 macrophage‐like cells. (A) Phagocytosis of monocytes differentiated towards M1 and M2 macrophage‐like cells, in the absence (M1 and M2) or presence of hAMTCs (M1‐hAMTCs and M2‐hAMTCs) or CM (M1‐CM and M2‐CM) was evaluated by flow cytometry. Resulting cells from different co‐culture conditions were incubated with fluorescent latex beads at 37 °C (white histograms) or 4 °C (control, grey histograms) for 6 and 24 h. The data shown are representative of at least three individual experiments. Numbers represent the mean fluorescence intensity (MFI) of uptake at 37 °C for each condition (mean ± SD; *p < 0.05 vs control M1 or M2). (B) The cytokines/chemokines were quantified in supernatants from monocytes differentiated under M1 or M2 culture conditions, in the absence (control M1 and M2) or presence of hAMTCs (M1‐hAMTCs and M2‐hAMTCs) or CM (M1‐CM and M2‐CM). Supernatants from hAMTCs in the transwell system, cultured alone or in the presence of the factors used to induce M1 or M2 macrophage differentiation, and CM used to treat monocytes, were included as controls (see supporting information, Figure S1). The results are expressed as mean ± SD of three individual supernatants for each population (*p < 0.05, **p < 0.01, ***p < 0.001 vs control M1 or M2 macrophage‐like cells)

Next, we investigated the functional properties of macrophages differentiated in the presence of hAMTCs or CM in terms of phagocytic activity and cytokine and chemokine profile. In accordance with others (Verreck *et al.*, 2004), phagocytic activity (measured as the mean of fluorescence intensity in each cell) of M2 was greater than that of M1 macrophage‐like cells (Figure 2A), although no differences were observed in terms of the percentage of cells that have engulfed particles (6 h: M1 87% ± 6%; M2 97 ± 1.8%; 24 h: M1 97% ± 1.1%; M2 99 ± 0.3%). The presence of hAMTCs or CM under M1‐like differentiation conditions increased the phagocytic capacity of cells (Figure 2A), again suggesting that hAMTCs and CM may interfere with the acquisition of the M1 phenotype while promoting monocyte differentiation towards M2 macrophage‐like cells. Instead, we observed a reduction of phagocytic activity when monocytes treated under M2 conditions were cultured in the presence of hAMTCs or CM (Figure 2A).

Given the fundamental role of cytokines and chemokines in modulating macrophage functions, we measured pro‐ and anti‐inflammatory cytokines and chemokines in supernatants collected from monocytes differentiated towards macrophages in the absence or presence of hAMTCs or CM. Monocytes under M1 differentiation conditions exposed to hAMTCs or CM showed a significant decreased expression of almost all the pro‐inflammatory factors investigated, including IL‐1*α*, IL‐1*β*, IL‐12, IL‐8, TNF*α*, MIP1*α*, MIP1*β*, MIG, Rantes and IP‐10, and an increase of anti‐inflammatory cytokine IL‐10 (Figure 2B). Moreover, in monocytes cultured under M2 differentiation conditions, hAMTCs and CM further decreased the production of the inflammatory cytokines and chemokines, including MCP‐1 (Figure 2B).

### 3.3 Effect of hAMTCs or CM on macrophage ability to stimulate T cell function

**Figure 3:**
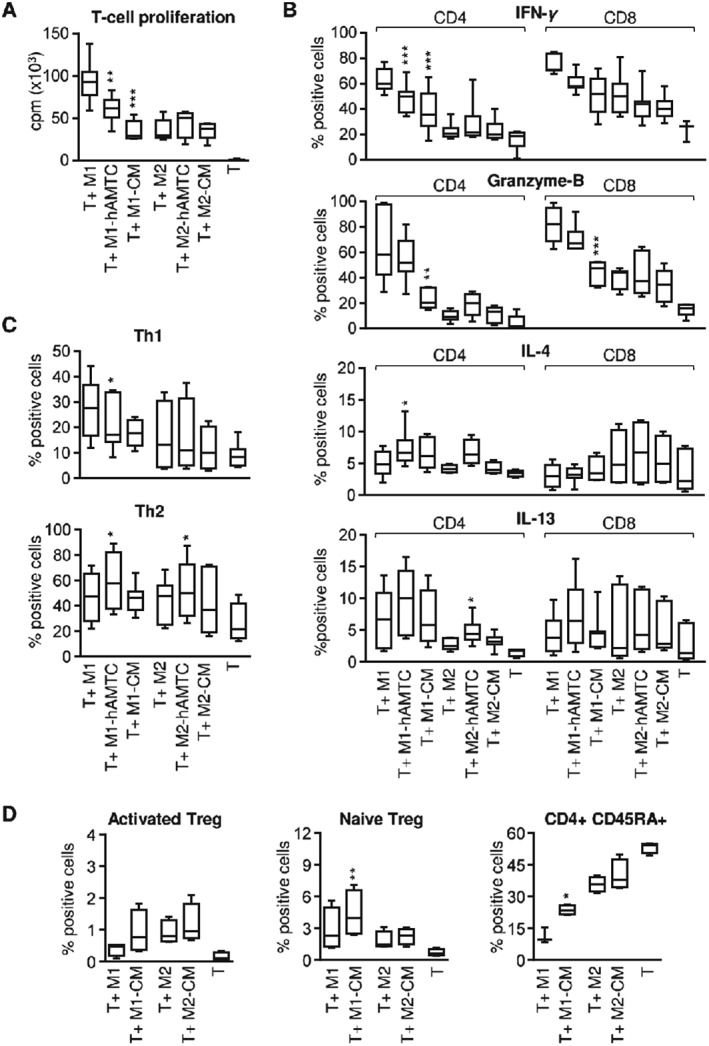
Effect of macrophage subsets on T cell proliferation, Th1/Th2 and Treg polarization and T cell cytokine expression. Monocytes differentiated towards M1 and M2 macrophage‐like cells, in the absence (control M1 and M2) or presence of hAMTCs (M1‐hAMTCs and M2‐hAMTCs) or CM (M1‐CM and M2‐CM), were collected after culture and used as stimulators of alloreactive T cells (T). (A) T cell proliferation was assessed by [^3^H]‐thymidine incorporation after 5 days of culture and expressed as counts/min (cpm). (B) The intracellular expression of IFN*γ*, Granzyme‐B, IL‐4 and IL‐13 was evaluated by flow cytometry on CD4^+^ and CD8^+^ gated T cells after 6 days of co‐culture. (C) Th1 and Th2 induction was evaluated by flow cytometry after 6 days of co‐culture and expressed as a percentage of CD4^+^ gated cells that were positive for CD119 and for GATA‐3, respectively. (D) Induction of Treg was evaluated by flow cytometry after 6 days of co‐culture, as a percentage of CD45RA^−^FoxP3^hi^CD25^hi^ for activated CD4^+^ Treg (left) and percentage of CD45RA^+^FoxP3^lo^CD25^med^ for naïve CD4^+^ Treg (middle). The percentage of naïve CD45RA^+^CD4^+^ T cells is shown in the right panel. The results represent the median ± IQR of at least five (A, C), four (D) and six (B) individual experiments. *p < 0.05, **p < 0.01, ***p < 0.001 vs T + M1 or T + M2)

To further assess the functional consequences of the macrophages obtained in the presence of hAMTCs or CM, we evaluated their ability to stimulate proliferation and cytokine production of allogeneic T cells. As expected, control M1 but not M2 macrophage‐like cells induced strong T cell proliferation (Figure 3A). However, monocytes differentiated towards M1 in the presence of hAMTCs or CM were poor inducers of T cell proliferation (Figure 3A). Moreover, we did not observe any differences in T cell proliferation between control M2 macrophage‐like cells and those differentiated in the presence of hAMTCs or CM (Figure 3A). Analysis of the intracellular expression of a panel of cytokines (including IL‐2, IL‐4, IL‐10, IL‐13, IL‐6, IFN*γ*, TNF*α* and Granzyme‐B) on T cells activated with M1 and M2 macrophage‐like cells, differentiated in the absence or presence of hAMTCs or CM, showed that M1‐hAMTCs and M1‐CM induced fewer IFN*γ*‐expressing CD4 T cells than control M1 macrophages (Figure 3B). Moreover, M1‐hAMTCs and M2‐hATMC increased the percentage of IL‐4‐ and IL‐13‐expressing CD4 T cells, respectively (Figure 3B). Interestingly, exposure of T cells to M1‐CM significantly decreased the percentage of CD8 and CD4 T cells that express Granzyme‐B (Figure 3B). No differences were observed in the percentage of cells expressing the other cytokines investigated (IL‐2, IL‐10, IL‐6 and TNF*α*) in either CD4^+^ or CD8^+^ T cell populations exposed to macrophages generated in the presence or absence of hAMTCs or CM (data not shown). The effects on T cell proliferation and cytokine expression were not due to M1‐CM and M1‐hAMTCs induction of T cell death (% cell viability, 85.8 ± 5.80% for T1 + M1; 85.9 ± 4.48% for T + M1‐hAMTCs; 84.9 ± 3.01% for T + M1‐CM; mean ± SD). Overall, these results indicate that hAMTCs or CM have a profound effect on monocyte differentiation, leading to macrophages with a reduced capacity to activate Th1 CD4^+^ and T1 CD8^+^, and possibly to induce Th2 CD4 T cells. To further confirm this hypothesis, we measured the expression of Th1 marker CD119 (IFN*γ* receptor), Th1‐associated chemokine receptor CXCR3 (CD183) and the Th2 transcription factor GATA‐3. Monocytes previously differentiated towards M1 in the presence of hAMTCs or CM induced a lower percentage of CD119^+^ (Figure 3B) and CD183^+^ (data not shown) T cells in the co‐cultures compared to the control M1. Reduction of the Th1 cell population was accompanied by a significant increase in the percentage of GATA‐3+ Th2 cells after stimulation with M1‐hAMTCs (Figure 3B). Whereas M2‐hAMTCs or M2‐CM did not affect the polarization of Th1 cells (Figure 3B), we observed a significant increase in the percentage of GATA‐3^+^ Th2 cells in the presence of M2‐hAMTCs as stimulators (Figure 3B).

Interestingly, flow‐cytometry analysis revealed the presence of Th1 (CD183 or CD119) and Th2 (GATA‐3) double‐positive T cells, indicating the induction of Th1/2 hybrid cells by macrophages *in vitro* (see supporting information, Figure S2B). Recently, the plasticity of the Th cell lineage has been discussed (Murphy and Stockinger, 2010) and the Th1/2 hybrid phenotype was observed both *in vitro* (Antebi *et al.*, 2013; Fang *et al.*, 2013) and *in vivo*, where stable hybrid Th1/2 cells (described as Tbet^+^GATA3^+^‐expressing CD183) were found to develop naturally in mice in response to parasites (Peine *et al.*, 2013) or viruses (Hegazy *et al.*, 2010).

### 3.4 Increased T cell polarization towards Tregs driven by macrophages generated in the presence of CM

To further obtain insight on how hAMTCs could affect the functions of macrophages, we investigated the impact of macrophage subsets on the polarization of CD4^+^ T cells towards regulatory T (Treg) cells. Since we obtained similar results from experiments with hAMTCs and CM, for subsequent experiments we used macrophages differentiated only in the presence of CM. Because human FoxP3‐expressing T cells are functionally heterogeneous and include suppressive and non‐suppressive T cells (Miyara *et al.*, 2009), we used the expression of FoxP3, CD25 and CD45RA to identify suppressive, activated Treg as CD45RA^−^FoxP3hi CD25hi CD4^+^ T cells; gating strategy is shown in Figure S2C (see supporting information). Although the differences were not statistically significant, the exposure of purified T cells to M1‐CM, M2 and M2‐CM increased the percentage of CD45RA^−^FoxP3hiCD25hi‐activated Treg cells in co‐culture in comparison to that of control M1‐macrophage‐like cells (Figure 3D, left). Interestingly, M1‐CM, but not M2‐CM, significantly increased the percentage of the naïve CD45RA^+^ subpopulation in CD4 T cells in comparison to their respective controls (Figure 3D, middle). Among T cells with a naïve phenotype, naïve Treg cells, defined as CD45RA^+^FoxP3lowCD25med CD4^+^ T cells, have been reported to possess potent suppressive functions (Miyara *et al.*, 2009). M1‐CM induced a significant increase of naïve Treg, compared to that induced by control M1‐macrophage like cells (Figure 3D, right). Altogether, the presence of CM during the differentiation of monocytes towards M1 generated macrophages with the ability to sustain the proportion of T cells with a naïve phenotype (CD45RA^+^ cells) and to drive their polarization towards a suppressive Treg phenotype.

### 3.5 Mechanisms involved in the CM‐induced macrophage phenotype

**Figure 4:**
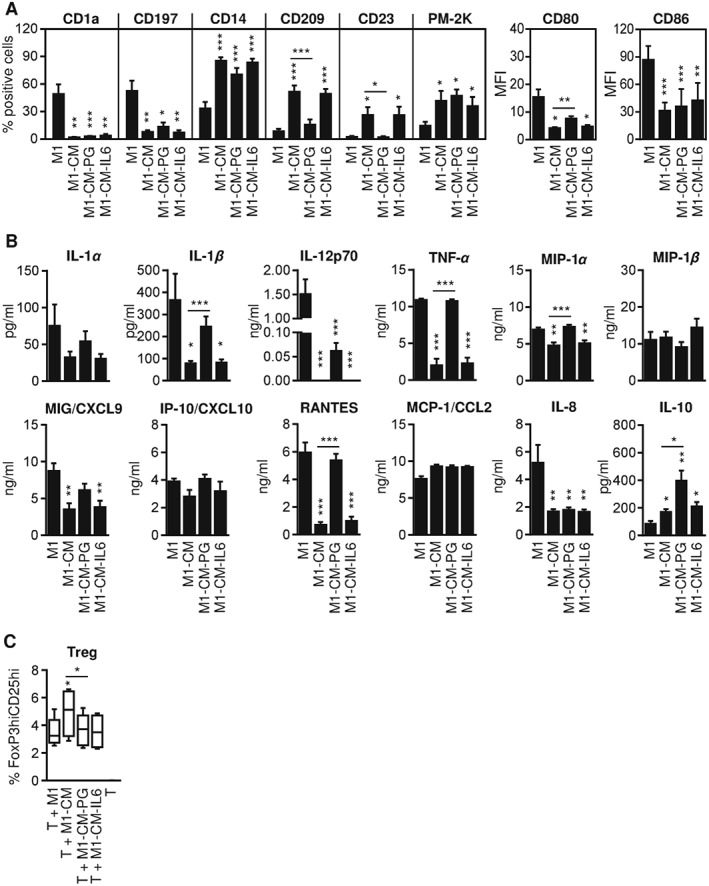
Effect of prostaglandins and IL‐6 on CM‐induced macrophage phenotype and function. Monocytes were differentiated under M1 conditions in the absence (M1) or presence of CM (M1‐CM), prostaglandin‐depleted CM (M1‐CM – PG) or IL‐6 blocked CM (M1‐CM – IL‐6). (A) Cell markers were measured by flow cytometry and expressed as mean ± SD (four individual experiments) of the percentage of positive cells (for CD1a, CD197, CD14, CD209, CD23 and PM‐2K) or of the mean fluorescence intensity (MFI; for CD80 and CD86). (B) Cytokine/chemokine quantification from supernatants of different co‐culture conditions: supernatants from CM, CM – PG or CM–IL‐6 used to treat monocytes were used as controls (see supporting information, Figure S1); the results are expressed as mean ± SD of six individual supernatants for each population. (C) Activated Treg cells were evaluated by flow cytometry after 6 days of co‐culture and expressed as a percentage of CD4^+^CD45RA^−^FoxP3^hi^CD25^hi^ cells; the results shown are median ± IQR of four individual experiments. *p < 0.05, **p < 0.01, ***p < 0.001 vs control M1 or vs M1‐CM when indicated

The effects observed by CM from the culture of unstimulated hAMTCs indicates that these cells constitutively secrete factors able to act on monocytes. We have previously shown that CM contains different prostanoids (such as PGD2, PGF2a and PGE2) and high levels of IL‐6 (Rossi *et al.*, 2012), both of which are partially responsible for the inhibition of T cell proliferation (Rossi *et al.*, 2012). Moreover, different studies have reported that PGE2 and IL‐6 contribute to differentiation towards M2‐like macrophages (Nemeth *et al.*, 2009; Maggini *et al.*, 2010; Eggenhofer and Hoogduijn, 2012; Abumaree *et al.*, 2013; Anderson *et al.*, 2013). We therefore investigated whether prostanoids and IL‐6 play a role in the shift from M1 differentiation towards an anti‐inflammatory M2 profile induced by CM. To this aim, we specifically inhibited the production of prostaglandins (PGs) in hAMTCs cultures using indomethacin, a cyclooxygenase inhibitor, and we used this medium (CM – PG) to test its effect on monocyte differentiation towards M1. As expected, we found a significant decrease of PGE2 in CM after indomethacin treatment (716.2 ± 198.7 ng/ml in CM; 0.7 ± 0.2 ng/ml in CM + indomethacin). In parallel, in order to block IL‐6, we differentiated monocytes with CM in the presence of neutralizing antibodies against IL‐6 and its cell surface heterodimeric receptor complex, consisting of the ligand binding subunit (IL6R*α*) and the signal transducing subunit gp130 (Murakami *et al.*, 1993). During monocyte differentiation, PG‐depleted CM was not able to induce M2 markers CD209 and CD23 and did not efficiently reduce CD80 expression to the extent of CM (Figure 4A). In contrast, blockade of IL‐6 was not able to reverse the effect of CM on any of the markers studied (Figure 4A). In accordance with these findings, PG depletion, but not IL‐6 blockade, reversed the inhibitory effect of CM on the production of several cytokines and chemokines by differentiated M1‐like macrophages, such as IL‐1*β*, TNF*α*, RANTES, and MIP‐1*α* (Figure 4B). However, the production of other cytokines (IL‐12p70, IL‐8) was not affected by the depletion of PG from CM, and the production of IL‐10 increased (Figure 4B). Overall, these data indicate that prostaglandins, but not IL‐6, seem to significantly contribute to the CM‐mediated modulation of macrophage differentiation. However, with the exception of its effect on the induction of Treg cells, neither PG nor IL‐6 play a major role in the functionality of M1‐CM cells. Thus, neither PG depletion nor IL‐6 blocking affected the phagocytic activity shown by M1‐CM (see supporting information, Figure S4A) or the low stimulatory effect of M1‐CM on T cell proliferation (Figure S4B) and Th1 differentiation (Figure S4C, D). In contrast, the absence of PG or IL‐6 signalling in CM reversed the effect of M1‐CM in the generation of Treg cells (Figure 4D).

### 3.6 Regenerative capacity of macrophages generated in the presence of CM in wound‐healing assays

**Figure 5:**
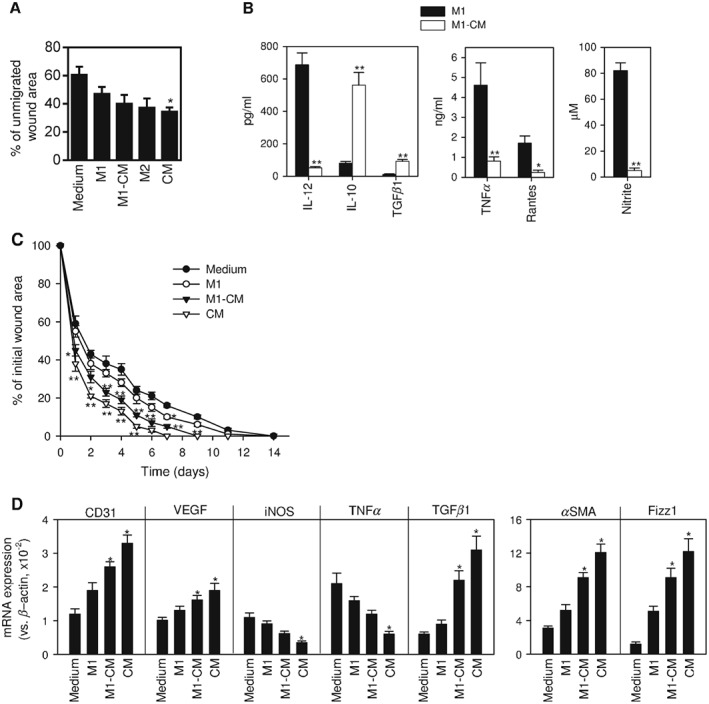
Effect of macrophage subsets in wound healing. (A) *In vitro* scratch wound‐healing assay was conducted with human fibroblasts plated in complete RPMI medium, used as control (medium), or with human monocytes differentiated under M1 conditions in the absence (M1) or presence of CM (M1‐CM), or under M2 conditions (M2), or with CM. The amount of wound was measured immediately after scratching (time 0) and 22 h after fibroblasts were scratched; the results are shown as mean ± SE of the percentage of remaining area of each condition, from five independent experiments; *p < 0.05 vs control medium‐treated fibroblasts. (B) Murine bone marrow‐derived macrophages were activated in the absence (M1) or presence (M1‐CM) of CM from hAMTCs; culture supernatants were assayed for cytokine levels by ELISA and for the content of nitrite (as an indirect measurement of the iNOS activity); the results are shown as mean ± SEM of 12 cultures; *p < 0.05, **p < 0.01 vs M1. (C) Murine macrophages (M1 or M1‐CM) or CM were injected into diabetic mice subjected to surgical skin wounding on the back: injection of complete DMEM was used as a control (Medium); the results show time course of wound closure after surgery and are expressed as a percentage of the original wound area; mean ± SE of eight mice/group, performed in two independent experiments; *p < 0.05, **p < 0.01 vs controls treated with DMEM. (D) Gene expression of inflammatory, angiogenic and macrophage markers in wound granulation tissue isolated 6 days after surgery; data are expressed as mean ± SE of four mice/group; *p < 0.05 vs controls treated with DMEM

We have shown that CM from hAMTCs was able to interfere with acquisition of the inflammatory M1 phenotype while promoting monocyte differentiation towards anti‐inflammatory, M2 macrophage‐like cells. To provide insight into the regenerative capacity of these cells, we applied them in a scratch wound assay and in a model of skin wound healing in diabetic mice. In the *in vitro* assay, confluent fibroblasts were scratched with a micropipette tip to create an artificial wound and then M1, M1‐CM, CM or M2 were added to the culture. Quantification of the unmigrated area revealed that the presence of macrophages and CM favours wound closure compared to fibroblasts alone, showing the following order of efficiency: CM > M2 > M1‐CM > M1 (Figure 5A).

Abnormal wound healing is a common complication of diabetes that leads to chronic skin ulcers and is caused by persistent pro‐inflammatory M1 macrophages recruited to the granulation tissue and insufficient angiogenesis (Schultze *et al.*, 2015). It has been suggested that a rebalance of M1/M2 macrophage phenotypes could contribute to accelerated healing in diabetic wounds. We generated mouse M1 macrophages differentiated from bone marrow precursors in the absence or presence of CM obtained from hAMTCs, and we used them in an established skin wound model in STZ‐induced diabetic mice. First, we investigated whether CM could generate a phenotype in murine cells similar to that observed in human monocytes. As expected, the presence of CM during the differentiation of macrophages in an M1‐driven environment resulted in macrophages that produced low levels of inflammatory cytokines (IL‐12, TNF*α*, RANTES) and nitric oxide (indicative of iNOS activity), and increased levels of IL‐10 and TGF*β*1 (Figure 5B). The systemic injection of mouse M1‐CM into diabetic mice with serial full‐thickness incisional wounds in the back significantly accelerated the closure of the ulcers compared to M1‐treated or untreated animals (Figure 5C). Interestingly, local administration of CM around the skin wound showed an even higher therapeutic effect (Figure 5C), suggesting that CM could induce the generation of M2‐like macrophages *in vivo*. Finally, we measured local wound expression of factors related to progression or healing. Treatment with M1‐CM or CM alone increased gene expression of *CD31* and *VEGF*, two factors involved in angiogenesis, and of *TGFβ1* and smooth muscle actin (*SMA*), two factors involved in tissue repair (Figure 5D). In contrast, injection of CM significantly reduced the expression of inflammatory markers such as *TNFα* and *iNOS* (Figure 5D). Interestingly, treatment of diabetic wounds with M1‐CM or CM increased the expression of the M2 marker Fizz1 (Figure 5D), again supporting the presence or induction of M2 cells, respectively, in the healed wound.

## 4 Discussion

Since macrophages are key modulators and effectors in immune responses, their activation influences other cells of the immune system, either directly or indirectly. In particular, macrophages could direct full T cell polarization to CD4 Th1, Th2, Th17 or Treg and CD8 cytotoxic T cells (Mills, 2012). Therefore, they have gained interest not only in the study of the pathogenic responses but also in situations where immune response is a key mechanism, such as in the paracrine responses employed in regenerative medicine approaches. As for the lymphocyte system, a dichotomy has been proposed for macrophage activation – M1 and M2, which respectively describe the two major and opposing activities of macrophages. Even though it is clear that it is time for reassessment, generally speaking, pro‐inflammatory features are attributed to M1 macrophages and anti‐inflammatory, tissue‐healing features to M2 (Mills, 2012).

Recently, different studies have demonstrated the potential of mesenchymal stromal cells (MSCs) from different sources to ‘educate’ macrophages to adapt an anti‐inflammatory, immune‐suppressive phenotype (Kim and Hematti 2009; Maggini *et al.*, 2010; Abumaree *et al.*, 2013; Anderson *et al.*, 2013).

Although it has previously been reported that hAMTCs act directly on monocytes (Magatti *et al.*, 2015, 2009) and that amniotic‐derived cells decrease the production of TNF*α* and IL‐6 cytokines in LPS‐stimulated monocytes (Li *et al.*, 2015), we here provided the first evidence that hAMTCs and their CM induce macrophage differentiation in the promonocytic U937 cell line or peripheral blood monocytes, in the absence of any other exogenous agents. Importantly, we demonstrated that hAMTCs or their CM favour the generation of macrophages with M2‐like features, even in a M1‐conditioned environment, consequently affecting their functions, such as higher phagocytosis. Moreover, there was a moderate increase of the anti‐inflammatory cytokine IL‐10 and decrease of several pro‐inflammatory factors. This cytokine/chemokines profile, associated with the reduced expression of co‐stimulatory molecules, rendered these cells poor inducers of T cell proliferation. Consistent with their lower stimulatory activity, macrophages generated in the presence of hAMTCs or their CM increase the percentage of naïve T cells expressing CD45RA, a marker expressed on undivided naïve CD4^+^ T cells that strongly decreases following T cell stimulation (Ma *et al.*, 2004). Moreover, macrophages resulting from monocyte differentiation under M1 culture conditions in the presence of hAMTCs and CM were less efficient in activating and/or inducing Th1 cells (CD4 T cells expressing INF*γ* and its receptor CD119), in line with their low expression of CD80 and low secretion of IL‐12 and CXC10, thus altogether not conducive to an inflammatory, Th1 micro‐environment. Of note, hAMTCs and CM modulate monocyte differentiation even under M2 conditions, leading to M2‐like cells with an even stronger anti‐inflammatory profile, characterized by a reduced expression of the co‐stimulatory molecule CD80, decreased secretion of several inflammatory chemokines and reduced phagocytosis activity. Phagocytosis mediates turnover of cell death in the body; it removes pathogens and cell debris, thus contributing to maintaining tissue integrity. However, under certain conditions, such as inflammation, in ischaemia and neurodegeneration, phagocytosis can exacerbate damage progression and contribute to disease (Neher *et al.*, 2012; Brown and Neher, 2014). The ability of AMTCs or CM to influence and limit phagocytosis in M2‐like cells might be a key pathway to conferring protection, and also suggests their possible application in diseases where there is an altered M2 function. This hypothesis is supported by recent findings showing that MSCs contribute to the resolution of certain diseases mediated by excessive M2 expression, such as that observed in animal models of allergic asthma (Mathias *et al.*, 2013), skin fibrosis (Qi *et al.*, 2014), sepsis (Nemeth *et al.*, 2009) and traumatic brain injury (Zanier *et al.*, 2014).

In addition, macrophages generated under M1 conditions in the presence of hAMTCs and CM induced the emergence of activated and naïve Treg cells with potential suppressive activity. We previously reported the capacity of CM from hAMTCs to increase the percentage of CD4^+^FoxP3^+^ cells in mixed lymphocyte cultures, but not the expression of FoxP3 in purified T cells (Pianta *et al.*, 2015), suggesting that CM‐hAMSC does not act directly on T cells inducing Treg cells, but very likely acts indirectly on other immune cells. Our study points to monocytes ‘educated’ to M2‐like macrophages as potential indirect inducers of Treg cells by hAMTCs and CM.

Furthermore, we have demonstrated that M1 macrophages generated in the presence of hAMTCs or CM induced CD4 and CD8 T lymphocytes with reduced expression of proteinase granzyme‐B. Cytotoxic Granzyme‐B plays an important role in cell apoptosis, extracellular matrix remodelling and in promoting inflammation, ultimately influencing cytokine production and cytokine processing from inactive to active forms (Hiebert and Granville, 2012). It has therefore been implicated in the pathogenesis of several diseases associated with impaired tissue healing and remodelling, chronic inflammation and the autoimmune response (Hiebert and Granville, 2012). Thus, the suppression of Granzyme‐B could be another mechanism by which hAMTCs exert their immunomodulatory/reparative effects *in vivo*.

Use of the transwell system and CM from the culture of unstimulated hAMTCs has indicated that hAMTCs could act on monocytes via constitutively secreted factors, without the need for cell–cell contact or activating stimuli. Amniotic cells have been shown to express a broad spectrum of factors that could account for the immunosuppressive action exerted on the differentiation of M1 macrophages, such as HGF (Kronsteiner *et al.*, 2011; Yamahara *et al.*, 2014) and prostanoids (PGD2, PGF2a and PGE2) (Rossi *et al.*, 2012), which have been shown to modulate the immune functions of monocytes/macrophages (Galimi *et al.*, 2001; Chen *et al.*, 2014) and contribute to differentiation towards M2‐like macrophages (Nemeth *et al.*, 2009; Maggini *et al.*, 2010; Anderson *et al.*, 2013). Moreover, hAMTCs also produce IL‐6, and IL‐10 (Rossi *et al.*, 2012), which, together with PGE2, have been described to induce M2 macrophages *in vitro* (Buechler *et al.*, 2000; Heusinkveld *et al.*, 2011; Kalinski, 2012). In this study, we show that prostaglandins, and not IL‐6, seem to be involved in the suppressive activity of macrophage differentiation, confirming previous studies indicating PGE‐2 as a relevant factors in macrophage differentiation (Nemeth *et al.*, 2009; Maggini *et al.*, 2010; Anderson *et al.*, 2013). However, the inhibition of prostaglandins did not completely reverse the CM inhibitory effect, indicative of a more complex mechanism. Indeed, in the presence of CM without prostaglandins, we obtained macrophages that, compared to those differentiated with control CM, did not express the M2 markers CD209 and CD23 but did express the M2 marker CD14 and the macrophage marker PM‐2K, in parallel with the reduced expression of M1 markers CD1a, CD197, CD80 and CD86. Moreover, these macrophages maintained higher phagocytic activity and were poor stimulators of T cell proliferation, further substantiating the M2‐like profile. Macrophages generated in the presence of prostaglandin‐depleted CM retained high levels of numerous pro‐inflammatory cytokines, such as IL‐1*β*, TNF‐*α*, MIP1‐*α*, MIG/CXCL9 and RANTES, with concomitant low levels of IL‐12 and IL‐8 and high anti‐inflammatory IL‐10. The M2 classification represents the extremes of macrophage activation states, and different subtypes of M2 macrophages (M2a, M2b, M2c and M2d) have been described, based on *in vitro* differentiation activators, macrophage marker expression and cytokine/chemokine production. M2 cells are generally characterized by low production of pro‐inflammatory cytokines, with the exception of M2b, which retain high levels of inflammatory cytokine (IL‐1, TNF and IL‐6) production with simultaneous high IL‐10 and low IL‐12 (Mantovani *et al.*, 2004). Thus, macrophages generated in the presence of prostaglandin‐depleted CM showed a profile similar to that of M2b cells. Consequently, prostaglandins do not seem to be key molecules for the switch from M1‐ to M2‐like macrophages, but rather they likely play important role in directing different functional states of M2 macrophages.

Importantly from a physiological and therapeutic point of view, we provide evidence that both CM and CM‐educated macrophages efficiently promoted skin wound healing. Macrophages play important roles in tissue integrity and host defence and coordinate each phase of wound healing. After a trauma or infection, the healing process is characterized by an inflammatory stage mainly orchestrated by inflammatory M1 macrophages, which induce production of pro‐inflammatory cytokines, chemokines and reactive oxygen species to eliminate invading pathogens or injurious initiating factors. Afterwards, M2‐like cells promote the progression from the inflammation phase to the tissue repair phase, restoring the damaged tissue architecture and integrity (Mills, 2012; Wermuth and Jimenez, 2015). Dysregulation of this process can result in several diseases, and excessive M1 activity has been found to play a major role in atherosclerosis, autoimmunity and other chronic inflammatory conditions. On the other hand, excessive M2 expression could lead to diseases such as allergy, fibrosis, chronic infections and cancer (Mills, 2012). Therefore, modulating macrophage activities has been designed as a new therapeutic approach to promote tissue regeneration (Sica and Mantovani, 2012). The fact that hAMTCs and CM could switch the differentiation of pro‐inflammatory M1‐macrophages into a M2 anti‐inflammatory/immune‐suppressive phenotype could reveal the beneficial effects observed in different *in vivo* models (Parolini *et al.*, 2011; Silini *et al.*, 2013). Indeed, macrophage‐mediated resolution has been observed after treatment with amniotic‐derived cells or MSCs from different sources in liver fibrosis (Manuelpillai *et al.*, 2012), multiple sclerosis (Liu *et al.*, 2012), lung fibrosis (Murphy *et al.*, 2012; Cargnoni *et al.*, 2014; Tan *et al.*, 2015, 2014), tendon lesions (Mauro *et al.*, 2016) and skin wound healing (Zhang *et al.*, 2010).

In the present study, we demonstrated that CM was also able to convert BM‐derived murine macrophages into a suppressive phenotype *in vitro*, characterized by high production of IL‐10 and TGF*β*1 and low production of inflammatory IL‐12, TNF*α*, RANTES and nitric oxide. It is noteworthy that CM accelerated *in vivo* skin wound closure when subcutaneously injected at the wound site. The CM‐mediated tissue repair was associated with the modulation of resident myeloid populations, characterized by reduction of the inflammatory environment and M1 macrophages (as demonstrated by reduced expression of TNF*α* and iNOS in the wound granulation tissue), and induction of murine M2 macrophages (Fizz1). Of note, we measured high expression of TGF‐*β*1, a factor that triggers fibroblast to myofibroblast transdifferentiation. Myofibroblasts are a unique population of MSCs capable of accelerating wound repair and which express *α*‐SMA (Abraham *et al.*, 2007), which we confirmed herein to be increased. Moreover, we observed increased expression of CD31 and VEGF, indicative of endothelial cell survival. Interestingly, we provided evidence that the injection of *ex vivo* CM‐modulated macrophages directly managed the wound‐healing regeneration. Transplantation of *ex vivo* educated macrophages has been recently suggested as a new innovative therapeutic approach for inflammatory disorders (Sica and Mantovani, 2012), such as spinal cord injury (Ren and Young 2013) or myocardium infarct (Leor *et al.*, 2006). We have previously demonstrated the protective role of adipose‐derived MSC‐educated macrophages transplanted in experimental colitis and sepsis (Anderson *et al.*, 2013), and in the present study we have shown the beneficial effect of CM‐educated macrophages in the resolution of wound healing, opening exciting possibilities for its future therapeutic use.

## 5 Conclusion

For the first time, in this study we showed that secreted factors from hAMTCs impact the generation of both M1‐ and M2‐macrophage‐like cells from peripheral blood monocytes by switching the differentiation of classical activated/pro‐inflammatory M1‐macrophages into M2‐like anti‐inflammatory/regulatory macrophages, and inducing regulatory/wound‐healing M2‐macrophage like cells with an enhanced anti‐inflammatory profile. Importantly, our study may help to clarify the underlying mechanisms of the beneficial activity observed *in vivo* after their administration, and provide evidence of the beneficial effect of CM and CM‐educated macrophages in tissue repair. In addition, these results could be relevant for the development and definition of the applications of hAMTCs and CM for the resolution of pathologies based on altered macrophage activation.

### Conflict of interest

The authors declare no conflicts of interest.

### Author contributions

M.M. participated in the design of the study, performed *in vitro* experiments, acquired and analysed the data and drafted the manuscript; E.V. and S.D. carried out *in vitro* experiments and acquired the data; M.Caro carried out *in vivo* studies; M.Caruso participated in the design of the study and in drafting the manuscript; A.S. analysed the data and participated in drafting the manuscript; M.D. designed and performed *in vivo* experiments and participated in drafting and revising the manuscript; and O.P. conceived the study, participated in its design, supervised the research, participated in drafting and critically revised the manuscript. All authors read and approved the final version of the manuscript.

## Supporting information


**Figure S1.** Cytokine and chemokine levels in supernatants from hAMTCs and CM used to treat monocytes. Cytokine and chemokine levels for supernatants used in the experiments shown in Figures 2 and 4. Specifically, these include supernatants from hAMTCs in the transwell system cultured alone (hAMTCs), or in the presence of the factors used to induce the M1 (GM‐CSF, INF‐*γ*, LPS) or M2 (M‐CSF, IL‐4, LPS) macrophage differentiation (hAMTCs (M1) and hAMTCs (M2), respectively), and CM, prostaglandin‐depleted CM (CM – PG), and IL‐6‐blocked CM (CM–IL‐6). Results are expressed as mean ± SD of at least 3 individual supernatants
**Figure S2.** Representative gating strategy of T cell phenotype analysis. Viable cells were gated on the basis of Zombie fluorescence and morphological (forward and side scatter) parameters. Then, T cells were distinguished from macrophages as CD3 positive cells. (A) Intracellular IL‐13, IL‐4, Granzyme‐B, and IFN‐*γ* expression on CD4 and CD8 T cells. (B) Th1 and Th2 analysis was evaluated on CD4 gated cells as CD183 or CD119 expression for Th1 and GATA‐3 expression for Th2 cells. Cells double positive for CD183 and GATA‐3 are shown (Th1/2 hybrid cells). (C) Activated Treg were identified in the CD4+ and CD45RA‐gate as FoxP3^hi^CD25^hi^ (a). Naïve Treg were identified in the CD4+ and CD45RA+gate as FoxP3^lo^CD25^med^ (b). The histograms show the fluorescence intensity of FoxP3 and CD25 of (a) and (b)
**Figure S3.** Effect of hAMTCs and CM on U937 phenotype and phagocytosis. U937 cells were induced to differentiate towards macrophages through culture with PMA (U937+PMA). Alternatively, U937 were cultured alone (U937), or in the presence of hAMTCs (U937‐hAMTCs) or CM (U937‐CM). Phenotype (A) and phagocytosis (B) of U937 resulting from different co‐culture conditions were evaluated by flow cytometry. (A) Cells were incubated with anti‐human monoclonal antibodies (white histograms) or isotype‐matched IgGs (control, grey histograms). The histograms shown are representative of at least 3 individual experiments. Numbers represent the mean value ± SD of the percentage of positive cells for each marker (*p < 0.05, **p < 0.01, ***p < 0.001 vs U937). (B) Cells were incubated with fluorescent latex beads at 37°C (white histograms) or at 4°C (control, grey histograms) for 6 h and 24 h. The mean fluorescence intensity (MFI) and the percentage (%) of uptake at 37°C are indicated. The data shown are representative of at least 3 individual experiments
**Figure S4.** Effect of prostaglandins and IL‐6 on macrophage phagocytosis and macrophage‐induction of T cell proliferation, Th1/Th2 polarization, and T cell cytokine expression. Monocytes were differentiated under M1 conditions in the absence (M1) or presence of CM (M1‐CM), prostaglandin‐depleted CM (M1‐CM – PG), or IL‐6 blocked CM (M1‐CM – IL‐6). (A) Phagocytosis was evaluated by flow cytometry after cell incubation with fluorescent latex beads at 37°C for 6 h and 24 h. Bar graphs represent the mean value ± SD of MFI of bead uptake from 4 individual experiments. (B‐D) Purified T cells were co‐cultured with macrophages previously generated M1, M1‐CM, M1‐CM – PG or M1‐CM – IL‐6. (B) T cell proliferation was assessed by [^3^H]‐thymidine incorporation after 5 days of culture and expressed as counts per minute (cpm). (C) Induction of Th1 cells was evaluated by flow cytometry as percentage of CD4+ gated cells positive for CD183. (D) The intracellular expression of IFN‐*γ*, and Granzyme‐B, was evaluated by flow cytometry on CD4+ and CD8+ gated T cells. The results shown are the median ± IQR of 4 individual experiments. *p < 0.05, **p < 0.01, ***p < 0.001 vs M1

Supporting info item

Supporting info item

Supporting info item

Figure S4. Effect of prostaglandins and IL‐6 on macrophage phagocytosis and macrophage‐induction of T cell proliferation, Th1/Th2 polarization and T cell cytokine expression
